# Microstructure and Mechanical Performance Correlation in a Pulsed Laser Welded IN792 DS Alloy

**DOI:** 10.3390/ma19132704

**Published:** 2026-06-23

**Authors:** Giovanni Maizza, Peihong Cheng, Alessandra Varone, Roberto Montanari

**Affiliations:** 1 Department of Applied Science and Technology, Politecnico di Torino, 10129 Torino, Italy; 2 Department of Industrial Engineering, University of Rome “Tor Vergata”, 00133 Rome, Italy; peihong.cheng@students.uniroma2.eu (P.C.); alessandra.varone@uniroma2.it (A.V.); roberto.montanari@uniroma2.it (R.M.)

**Keywords:** pulsed laser welding, IN792 DS, Nano-IIT grid mapping, ISE-free property, loading secant stiffness rate, statistical deconvolution analysis

## Abstract

This study investigates the mechanical performance of a pulsed laser butt-welded IN792 DS joint and its relationship to its microstructure by means of grid nanoindentation. A new ISE-free (rate-derived) hardness parameter (H_R_) has been introduced to account for the local bulk elastoplastic behavior of the material in combination with the stable contribution of residual stress, thus overcoming the limitations of the current standard codes. It allows performance comparability between different welding experiments, materials, and joint configurations. It offers an alternate means to mechanically determine the HAZ width when microscopic and metallurgical methods fail to detect it. Moreover, the spectra of two independent indentation parameters have been utilized as an input within an iterative statistical deconvolution scheme to estimate the composition of the relevant phases present within the fused zone. While one parameter spectrum acted as a predictor in the first stage, the second one served as a corrector for the final estimation of the four detected phases, thereby self-validating the iteration procedure with 5% tolerance. The validity of phase estimation was first determined over the entire FZ and then at three levels of the weald seam (top, neck and bottom) for further validation. The results indicate that the γ-matrix and ultrafine fine/hard second phases in the fused zone amounted to 54% and 43% volume fractions, respectively. The associated deconvoluted mechanical performance, expressed in terms of E_IT_, H_IT_, and H_R_, corresponded to approximately 209 ± 4.5, 6.3 ± 0.2, 4.4 ± 0.1 and 224 ± 7.0, 6.7 ± 0.1, and 4.6 ± 0.1 GPa, respectively. A correlation between the estimated phases and the local mechanical performance via the conventional indentation parameter (H_IT_ and E_IT_) and the new H_R_ parameter in the three relevant regions of the fused zone was discussed while discerning the effect of cooling rate on precipitate size, heterogeneity, porosity, residual stresses, and grain orientation. Further validation studies on different sample geometries, materials and joint configurations are needed to confirm the generality of the proposed methodology.

## 1. Introduction

High-power beam technologies are currently enjoying an unprecedented boost in fusion welding research, and repair applications are expected to expand even more in the future to enhance more sustainable manufacturing and products. Nickel-based superalloy components and systems are central to many strategic industrial sectors (e.g., nuclear, chemical, aircraft, power plants, etc.). For several decades, the safe use of fusion welded structures in nickel-based superalloys has been inhibited because of their tendency to crack [1,2,3]. The latest laser beam welding (LBW) technologies have contributed toward significantly mitigating the problem by introducing a much smaller weld seam and narrower heat-affected zone (HAZ) [4,5,6]. Recent evidence has shown that the LBW technology could even outperform conventional fusion welding methods as a result of its producing fully penetrated, crack-free welded joints in thin plates [7,8,9,10] and, recently, in thick plates (up to 22 mm) [11].

During laser beam welding (LBW), a steep thermal gradient and rapid thermal cycles generate a complex, heterogeneous microstructure across the weldment. The initial grain orientation of the FZ was prevalently altered such that new grains elongated under the control of the local thermal gradient. The HAZ induced by LBW is usually quite narrow, due to the severe heat concentration and fast cooling with respect to its surrounding regions (e.g., heat stagnation), which, in turn, originates a severe stress/strain field [12,13]. Thus, assessment of the mechanical performance of welded joints is crucial to obtain knowledge and reveal the level of integrity and the bearing capability of joints for designed applications.

Many researchers have demonstrated that the indentation test is a valuable method for mechanical characterization of welded joints. Pham and Kim [14] developed a comprehensive study of the cross section of an SM490 steel joint by cross-linking a nano-IIT to discern the mechanical performance (in terms of indentation modulus, E_IT_, and indentation hardness, H_IT_) across the fused zone (FZ), heat-affected zone (HAZ), and base material (BM). High- and low-stiffness ferrite and a pearlite phase fraction can be distinguished based on statistical deconvolution analysis of indentation parameters. The microstructure composition can be identified based on their corresponding indentation properties. Dai and Lippold [15] performed a displacement controlled nano-instrumented indentation test (nano-IIT) at the preset displacement of 500 nm (which corresponded to ~30 mN peak load) and a micro-Vickers indentation test (100 g) to characterize a dissimilar F22 steel/625-alloy welded joint. The indentation properties were found to be not only sensitive to the microstructure constituents [14,15,16] but also to the stress/strain field [17,18,19] and grain orientation [20,21]. Ye et al. [22] investigated the grain size and γ′/γ″ precipitates in dissimilar IN718/IN713LC joints by means of micro-Vickers hardness, whereas Preuss et al. [23] reported the hardness, γ grain size, grain orientation, γ′ precipitate size, and volume fraction in the case of a welded RR1000 joint. However, in all studies, the regions investigated mechanically by either nano-IIT or micro-Vickers were limited to small regions or the FZ or HAZ. To the authors’ knowledge, no studies have reported on nanoindentation mapping for the mechanical characterization of the entire weld seam, nor have they attempted to establish correlations between mechanical performance and microstructure, including heterogeneity effects, crystal orientation, residual stress, etc.

The designed nano-IIT grid mapping methodology has proved to be particularly suitable for the characterization of highly heterogeneous microstructures and for the discrimination of the boundaries between the FZ and the HAZ. However, caution should be taken when using general indentation techniques over the nano- to micro-scale range, as strong indentation size effect (ISE) impede comparisons between indentation properties [24] measured at self-similar indentations under different peak loads [25]. For this reason, the interpretation of nanohardness measurements is challenging and it is difficult to directly compare them with others’ results.

The present work has been conducted to determine the mechanical performance of a butt-welded (2 mm thick plate), directionally solidified (DS) IN792 superalloy, produced by pulsed LBW, and its correlation with its microstructure through a novel instrumented indentation methodology. Previous studies [9,26,27] that inspired this work [9,26] focused on the feasibility of welding the IN792 DS superalloy using LBW and electron beam welding (EBW) by analyzing in detail the microstructure composition of the FZ, HAZ and BM as a function of the preheating temperature and scanning rate. The as-welded (DS) IN792 alloy joint was free from visible cracks after pulsed LBW [9]. The phase fraction of γ′ was found to strongly depend on the preheating temperature and scanning rate. Under a preheating temperature of 200 °C and a scanning rate of 1.5 m/min, about 50% γ′ was detected in the FZ by image analysis [27]. This phase consisted of nanocrystalline particles between 20 and 40 nm in size. The fraction of the carbide phase was undetectable by X-ray diffraction because of the small amount of these particles. It was finally determined that the remaining 50% was a mixture of prevalent γ with some residual carbides.

This paper aims to expand the previous work on the pulsed LBW (DS) IN792 alloy joint using a laser and preheating parameters identical to those reported in ref. [9] by developing an integral methodology which allows estimation by statistical means of the microstructure composition across the FZ using as an input the measured two-dimensional map of the mechanical performance (in terms of the three extracted indentation parameters H_IT_, E_IT_ and H_R_) while drawing up a final correlation between them.

## 2. Materials and Methods

### 2.1. Material and Welding Process

**Figure 1:**
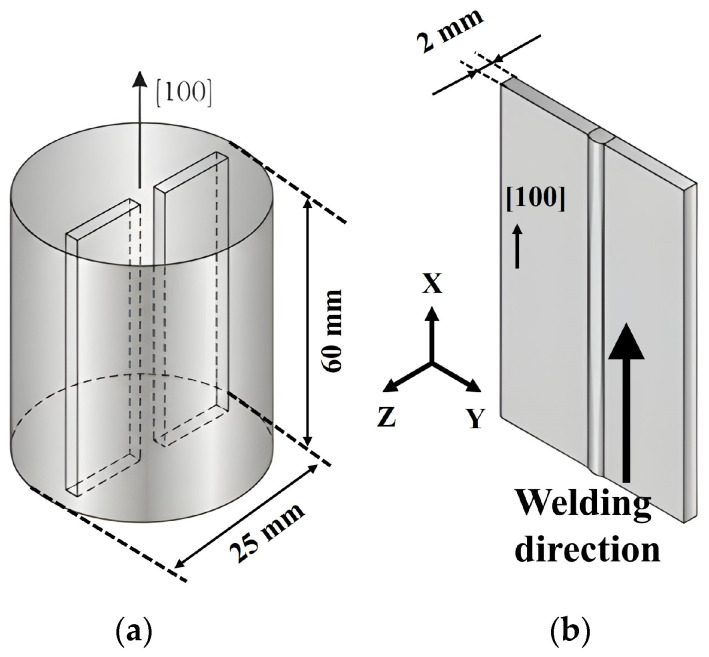
Original [100] DS ingot. (**a**) Cutting of the two plates along the [100] direction. (**b**) Butt-welding of the two plates along the [100] direction.

The nominal chemical composition of the investigated IN792 alloy is listed in Table 1. A [100] directionally solidified (DS) ingot (see Figure 1) was solubilized in a vacuum (1120 °C/2 h), then held at 845 °C/12 h and finally cooled in air to ambient temperature. Two thick plates (2 mm) were sliced by means of spark-erosion along the solidification direction and then butt-welded by pulsed LBW [9]. The welding (longitudinal, X) direction was parallel to the [100] crystal direction. The LBW parameters used are summarized in Table 2. More details on the experimental part of the experiment can be found elsewhere [9]. Assuming a negligible individual fraction of carbide phase in the FZ of the (DS) IN792 alloy joint (e.g., a 1–2% volume fraction in typical nickel-based superalloys [28,29]), we may assume for computational convenience that the carbide phase is combined with the more similar (50% fraction of) γ′ precipitate phase, as both share a similar metallurgical evolution and strengthening mechanism during welding, despite their respective particle sizes being quite different, e.g., hundreds of micrometers for the carbides and 20–40 nm for the γ′.

### 2.2. The IIT Methodology: Indentation Parameters

Automatic IIT conducted at the nanoscale (nano-IIT) via a grid mapping technique permits a detailed mechanical probing of highly heterogeneous materials, such as those observed in welded joints. Moreover, it can accurately detect the boundaries between the FZ, HAZ, and BM. This represents a unique advantage, especially when the HAZ width is very narrow, as in the case of nickel-based superalloys.

**Figure 2:**
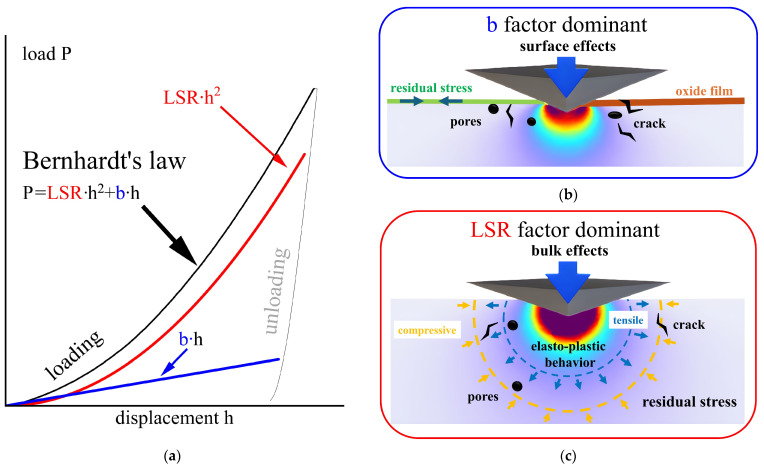
Bernhardt’s law indentation curve and inherent material response upon indentation. (**a**) Bernhardt’s law indentation curve. (**b**) Superficial mechanical response of the material with factors and anomalies acting at shallow penetration depth (superficial performance). (**c**) Elastoplastic response at large penetration depth with residual stress effects (bulk mechanical performance).

A new indentation parameter, called the loading stiffness rate (LSR), has been introduced to help discriminate the presence of RS in welded joints [30]. Its presence can be estimated by fitting the experimental indentation curve (IC) on loading with Bernhardt’s law, that is, F = LSR × h^2^ + b × h [31] (Figure 2a).

Various researchers have reported that Bernhardt’s law is more accurate than the standard power law for a variety of engineering materials [32,33]. LSR is an elastoplastic indentation (nearly ISE-free) parameter that is linked to the parabolic portion of the experimental IC (under indentations with pointed indenters). Its determination is made upon considering the last portion of the loading curve (Figure 2a). The linear term (b) in Bernhardt’s law measures the strength of the surface or subsurface anomalies that are responsible for any deviation from the parabolic law at the early stage of indentation [34]. These anomalies may be ascribed to various individual or a combination of factors, such as superficial oxides, voids, cracks, indenter bluntness, and/or superficial residual stress (Figure 2b). Indentation size effect phenomena, which naturally emerge at the beginning of indentation (Figure 2b), can also be embodied in the linear term [33,34]. However, this linear term (b) does not contribute to the elastoplastic response of the material, and it can therefore be considered as a loss of applied load which does not contribute to the desired material strain hardening. The larger the portion of the loading IC described by the parabolic term is, the larger the volume of the material sensed elastoplastically and represented by the LRS (Figure 2c). The load (or depth) range covered by the estimated LSR inevitably affects the unloading contact stiffness (S_u_) but not any eventual relaxation phenomena that occur during the holding stage under the peak load. Since LSR is derived as a slope from the S_h_-h curve (Figure 2a), it retains a natural link to the E_IT_. In this work, it has been used as an alternate elastoplastic parameter, like indentation hardness, with an additional unique ISE-free feature. First, let us recall the definition of the projected area of contact, A = 4 × h_c_^2^ × tan(68) ≈ 24.5 × h_c_^2^, that applies to Vickers and Berkovich indenters. The contact depth, h_c_, in the formula is a central parameter in the standard code for the definition of the E_IT_ and H_IT_ [35]. However, its exact determination becomes doubtful in materials affected by RS. An alternative strategy to overcome such an obstacle is to replace h_c_ with h_m1_ (the maximum penetration depth at the loading stage), that is, the depth measured at the end of the loading curve (a parameter that is free of any assumptions). In the previous formula, by replacing h_c_ with h_m1_, we obtain a modified definition of the contact area, which hereinafter is denoted as the *effective area of contact*. This leads to a new hardness definition, the *on-loading hardness*, H_R_, which is expressed in terms of *the loading secant stiffness rate* (LSR):

H_R_ = LSR / 24.504 (1)

Following Equation (1), a few considerations arise: (a) as H_IT_ is a function of h_c_, the latter depends on the maximum penetration depth after holding and on any incipient unloading (h_m2_), and vice versa, H_R_ is a function of the maximum penetration depth on loading, h_m1_; (b) as the assumption of good compliance between an indenter and RS-affected materials is more realistic upon loading than upon incipient unloading, more theoretically based estimations can be made on the pyramidal indentation volume; (c) as h_m2_ ≤ h_c_ ≤ h_m1_, we can always expect H_IT_ ≥ H_R_ and even much greater values than that for an operating ISE. Nevertheless, as the difference between h_m1_ and h_c_ is not numerically significant for the materials at hand, under steady-state testing conditions the difference in hardness between H_IT_ and H_R_ is expected to be marginal. Moreover, we preferred to use a hardness index that was determined from pure measurable quantities rather than an empirically corrected h_c,_ which is blind (and thus misleading). In our experiment, H_R_ was not used to replace H_IT_ but instead to complement it, in combination with E_IT_, in order to discern the presence and, if possible, the effects of heterogeneous microstructures and RS induced in the pulsed LBW joint during rapid solidification cycling. Nano-IIT was here used to sense the mechanical (elastic, plastic, and strain hardening) response that occurs across three joint regions, namely, the top (TR), neck (NR), and bottom (BR).

### 2.3. The IIT Methodology: The Grid Mapping Strategy

We conducted automatic nano-IIT grid mapping using a Hit 300 device (Anton Paar GmbH, Graz, Austria) equipped with a Berkovich diamond tip.

**Figure 3:**
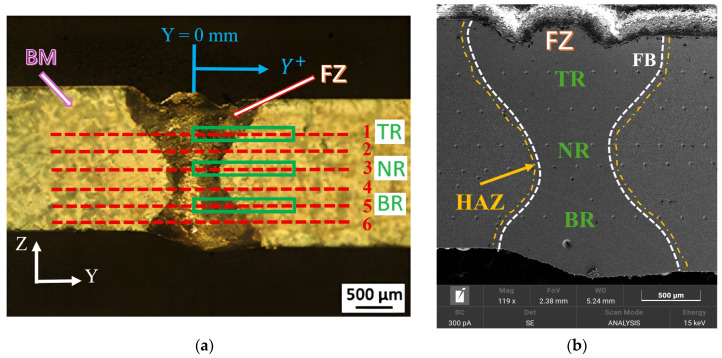
Nomenclature and definition of the relevant regions and the analyzed n-IIT performance lines. (**a**) Optical micrograph of the fully penetrated weld seam cross section (Y-Z plane) of the pulsed laser butt-welded as-cast IN792 DS joint (2 mm thick plates); the green rectangles define the **T**op, **N**eck, and **B**ottom **R**egions (denote as TR, NR, and BR) inspected by n-IIT; the red dashed lines define the six n-IIT performance lines; the FZ and BM regions are also denoted. (**b**) SEM image of the fully penetrated weld seam cross section (Y-Z plane, 119× magnification) with the relative nomenclature; the white dashed lines indicate the FB interfaces that separate the FZ from the HAZ; the outer boundaries are also sketched in orange dashed lines.

Prior to the test, the indented (Y-Z) cross-section surface of the LBW joint sample was mirror-polished mechanically using a conventional procedure. The grid mapping includes six horizontal lines along the transversal (Y) direction (see Figure 3a). The separation distance between the indentation lines was 0.25 mm. The lines included a total of 630 indents. Each line was divided into 21 groups, and there was therefore a total of 126 groups. Each group contained five equally spaced (0.030 mm) indentations, and each group was separated from the others by 0.075 mm. A trapezoidal load cycle, consisting of 5 mN/s loading/unloading rates, a 150 mN peak load, and a 10 s dwell time, was applied. Moreover, after the planned nano-IIT survey, the BM was tested at one point, at a distance of about 10 mm from the FZ, using macro-IIT (Zwick Röell ZHU 2.5, Vickers indenter; Ulm, Germany), with a load program that consisted of 4 identical trapezoidal cycles, a 100 N peak load, and a 30 s time span for loading, holding, and unloading, respectively. The deduced macro-IIT parameters defined the tensile-like properties of the starting plates. Figure 3a shows the typical hourglass shape of a fully penetrated weld bead produced in the laser keyhole mode. The vertical axis of the bead is tilted slightly to the left. Despite the slight asymmetry with respect to the *Z*-axis, the right side of the weld bead was investigated in more detail (Figure 3a). The left side was considered to provide complementary data that was used to support the measurements obtained from the portion on the right. The asymmetry between the top and bottom of the bead results from an unbalanced heat distribution across the plate thickness. The weld seam was virtually divided into three regions, as shown in Figure 3 (see the green blocks). These intercepted indentation Line 1 (TR), Line 3 (NR), and Line 5 (BR). The FZ was bound by the fusion boundary (FB), as shown in Figure 3b. The H_IT_, H_R_, and E_IT_ indentation parameters are represented, for each group, by their respective mean values and standard deviations.

### 2.4. Microstructure Composition: The Statistical Deconvolution Analysis

As the microstructure of the weld seam is unknown, except for in the few regions probed by microscopy, it is usually difficult to establish a quantitative correlation between the microstructure and its mechanical performance. Efforts have been made to extrapolate the volume fractions of the relevant phases that compose the microstructure by taking advantage of statistical deconvolution analysis [14,36,37] and using the phases detected by SEM and EBSD analysis as initial guesses. We associated three experimental frequency density distributions (in the form of histograms) to each measured indentation parameter (H_IT_, H_R_, and E_IT_). At least two of the three empirical distributions had to be independent to find a reliable solution. Pearson’s coefficient correlation analysis has shown that E_IT_ and H_IT_ (or H_R_) are not correlated [30]. Each experimental histogram exhibits characteristic peaks which may result from the mechanical response of distinct unknown phases. We applied this strategy to the FZ, in view of its intricate microstructure heterogeneity and its exceptional mechanical performance after pulsed LBW. Moreover, this portion of the weld seam was characterized by a sufficiently large number of indentation points, thereby making its inherent statistics sufficiently accurate. We defined a theoretical probability density function and forced it to approximate the experimental frequency density distribution by minimizing the sum of the squared deviations over the total observed values [36]. We then used the theoretical probability density function to perform a statistical deconvolution to extract a mixture of n gaussian distributions (or phases) which were considered to compose the FZ microstructure. Hence, the two experimental indentation parameters were assumed to vary, within each phase, according to a normal distribution. A judicious choice of the histogram peaks made the iteration procedure converge rapidly to the desired set of phases. In order to prevent the statistical optimization scheme converging toward a local minimum, the iteration scheme was started from a good initial guess equal to the number of relevant phases observed [9] by means of microscopy. The first-run solution provided the estimated phase fraction along with the arithmetic mean and standard deviation of the indentation parameter associated with each phase. The standard deviation measured the dispersion of the observed indentation parameter in each estimated phase. The statistical analysis was first performed on the entire FZ, using all the data collected within that zone, including 161 indents in total. Subsequently, it was repeated for the three distinct FZ regions, TR (35 indents), NR (16 indents), and BR (27 indents), using the subset of indentation parameters that were pertinent to each region.

## 3. Results

### 3.1. Microstructure Analysis

**Figure 4:**
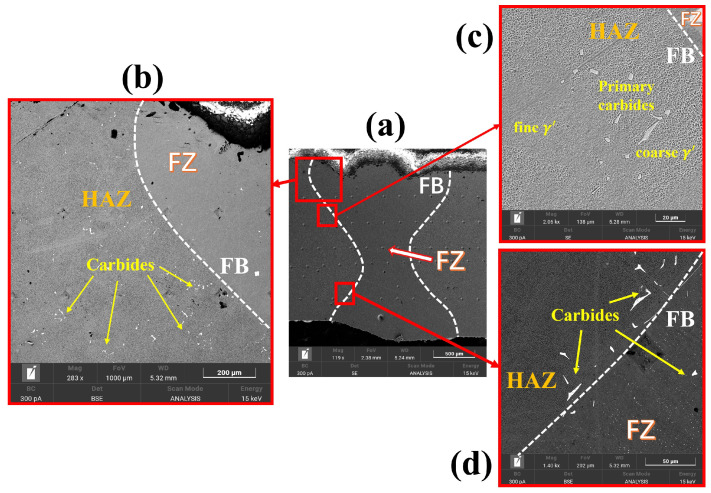
SEM micrographs of the microstructure features in the weld seam with details pertaining to near the fusion boundary. (**a**) The weld seam regions under investigation (119× magnification). (**b**) Backscattered electron (BSE) image of the distribution of coarse carbides within the HAZ (283× magnification), that is, of partially dissolved primary carbides that grew due to their vicinity to the high-temperature FB ((**c**) secondary electron image at 2000× magnification and (**d**) BSE image at 1400× magnification).

The FZ in Figure 3a shows a typical hourglass-shaped fully penetrated LBW joint with two slight undercuts on the top surface and a slight tilting of the weld seam with respect to the *Z*-axis. Too generous freedom of the sample in the clamping system favors its excessive misalignment upon welding. The hourglass shape results from the combined effects of recoil pressure and melt surface tension during fully penetrated LBW [38]. The cap width of the joint on the top surface (1.6 mm) is greater than that at the bottom (1.4 mm). Figure 4 shows the relevant microstructure features in the upper part of the weld seam, as observed by means of SEM. The inset indicates the analyzed areas depicted in Figure 4a–c. Figure 4a shows a backscattered electron microstructure image of the upper part comprising the FZ and HAZ. Various islands of carbides can be observed inside the HAZ. The microstructure of the FZ appears relatively fine compared to its adjacent HAZ. The latter is composed of separate coarse and fine islands, and it probably resulted from the full dissolution of eutectic phases on rapid heating, which then resolidified as a γ-matrix after rapid cooling (see Figure 4b). Figure 4c shows the magnification of a backscattered electron microstructure at the FB, close to the lower part of the weld seam (unetched phase). Different gray colors distinguish the matrix phase from the primary carbides. The FZ microstructure consists of dendritic and interdendritic zones; the former are rich in Fe, Ni, and Cr (γ former) elements, whereas the latter are rich in Al and Ti (γ′ former) elements, which contributed to the growth of carbides instead of the γ′ phase. It should be noted that, although the γ′ phase may derive from different routes during thermal cycling and thus form different shapes, all of these routes were treated here as unique phases.

**Figure 5:**
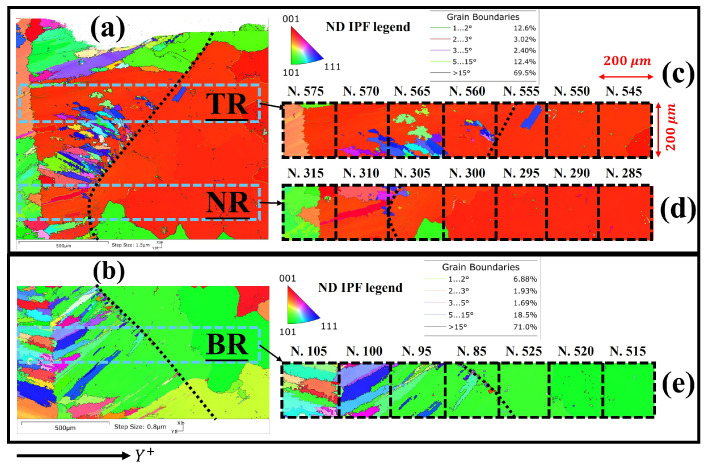
IPF//Z of the butt-welded DS IN792 alloy after pulsed LBW: (**a**) top neck section scanned with step size 1.5 μm; (**b**) bottom neck section of the weld seam scanned 0.8 μm; (**c**–**e**) indentation groups (identified by a number) at three relevant regions across the thickness. The dotted line refers to FB.

For convenience, the EBSD analysis was applied to the portion on the right of the weld seam with respect to the symmetrical *Z*-axis. Figure 5 shows the inverse pole figure (IPF) in the upper- and lower-half portions of the whole weld seam. The neck area was allowed to overlay the upper and lower portions in order to assess the influence of the selected step sizes used to scan the two parts of the weld seam on the final EBSD parameters. Accordingly, a step size of 1.5 μm was used for the upper portion, while one of 0.8 μm was used for the lower portion. The FB is drawn in a dotted black color in Figure 5a,b, whereas the cyan dashed-line rectangles delimit the three regions (TR, NR, and BR) used for the nano-IIT investigation. These three regions include the FZ, HAZ, and BM.

Figure 5a,b show the crystal orientation across the weld seam after rapid solidification as a result of the pulsed LBW of the IN792 DS alloy. The colors reflect the crystal orientations of the grains in the FZ, HAZ, and BM. The <001>//Z orientation, indicated in red, is dominant in the upper section of the weld. The original <001>//X (DS) direction of the cast alloy is preserved after solidification, which coincides with the easy growth direction of the solidified face-centered cubic (nickel-based) austenite in the FZ. Conversely, the <101> orientation consistently prevails in the lower section of the weld seam after continuous-wave LBW [39] and after electron beam melting [26] of the same IN792 DS alloy joints. This seems to be a common feature at the bottom of the keyhole during full penetration welding under high-energy-density laser melting [40]. Here, the <101>//Z microstructure orientation is present in the FZ and, surprisingly, also in the HAZ and BM. Sun et al. [40] observed that the curvature of the melt pool in the lower portion of the weld seam increased as the solidification proceeded, thus indicating dominant lateral migration of the FB. Moreover, the columnar grains across the FZ originate from the FB, and they grow epitaxially perpendicular to the FB along the maximum heat flux direction, while they elongate toward the weld centerline. Such grains exhibit a variety of higher crystal index orientations with higher stiffnesses. Although the epitaxial growth in the upper section originates from the <001> (red) γ-parent phase, it originates from the <101> (green) γ-parent phase in the lower section. However, the columnar grains seem to break down in some of the regions across the FZ and disappear in favor of the more dominant parent-phase crystal orientation (i.e., either <001> or <101> in the upper or lower sections, respectively). The observed narrow width of the HAZ is an indicator of the goodness of the pulsed LBW joint. Previous attempts to quantitatively detect the HAZ using optical microscopy and SEM, with and without EBSD, failed [9,41].

### 3.2. Nano-IIT Grid Mapping

**Figure 6:**
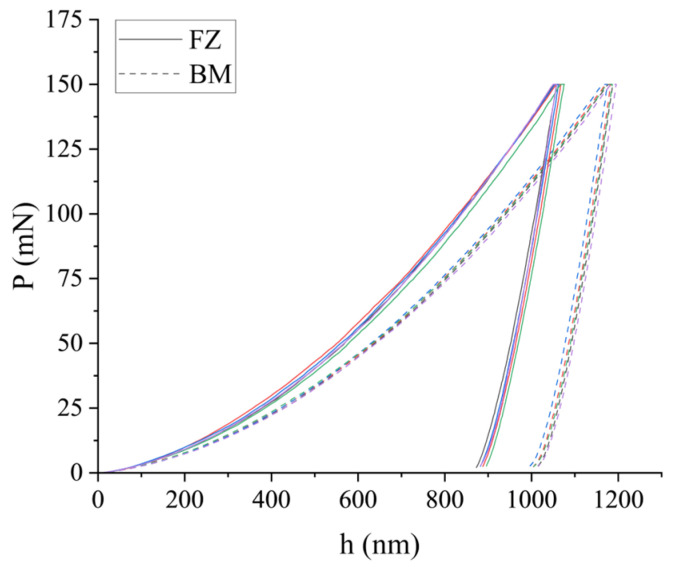
Typical nano-IIT curves measured at the center of both the FZ and the BM. The FZ exhibits greater hardness than the BM.

**Figure 7:**
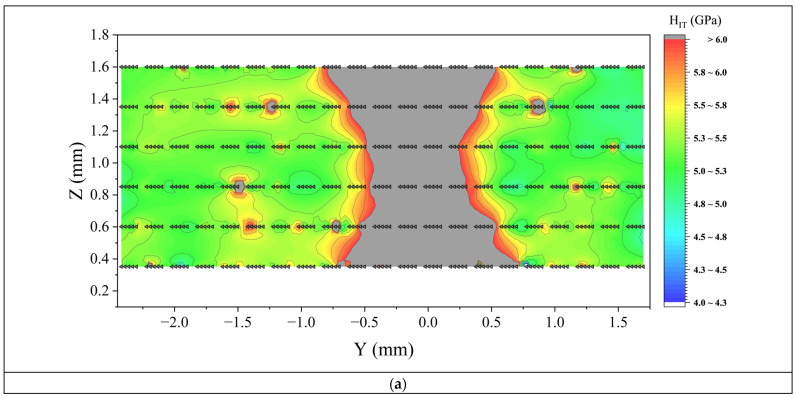

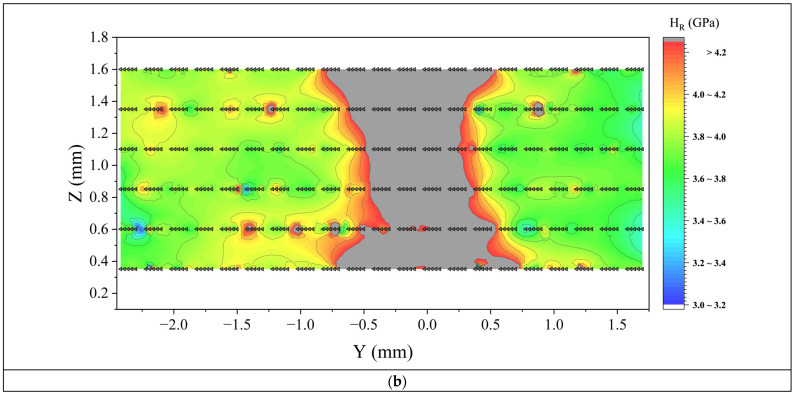
Spatial distribution of the nanohardness parameters after the pulsed LBW of the IN792 DS alloy plates in a butt joint: (**a**) H_IT_ distribution; (**b**) H_R_ distribution. Each triangle symbol represents a single indent; each five-indent array represents the group over which averages are computed.

Typical IC for the BM and FZ are shown in Figure 6. The FZ exhibits greater hardness than the BM. The extracted indentation parameters (H_IT_ and H_R_) across the weld seam cross section are shown as two-dimensional contour plots in Figure 7a,b. A *thin-plate smoothing spline* function of the MATLAB 2024 software [42] was used to smooth out the experimental data. The black color indicated in the color bar assigns the greatest hardness to localized coarse carbides. The FZ geometry detected in the nano-IIT and SEM images was observed to be consistent upon setting the hardness peaks to H_IT_ ≥6 GPa (Figure 7a) and H_R_ ≥4.25 GPa (Figure 7b). The difference between these hardness parameters reveals that ISE affects H_IT_, and any eventual surface anomalies may affect both parameters. Qualitatively, Figure 7b shows that the hardness variations about the FB line and around the coarse carbide precipitates look wider in the H_R_ plot than in the H_IT_ one.

**Figure 8:**
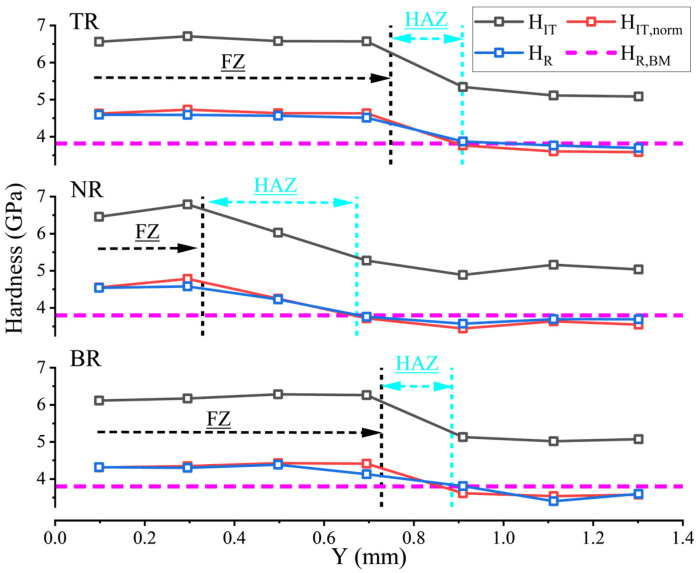
Profiles of the H_R_ hardness parameters (group-average value, blue line) at TR, NR, and BR; the normalized H_IT,norm_ (red line) and H_R,BM_ (pink dashed line) profiles have been added for comparison purposes. The vertical black and cyan dashed lines delimit the boundaries of the FZ and HAZ of the welded joint. Each square locates the indentation group over which averages have been computed.

The ISE-free nature of LSR was verified in the BM by comparing its respective values, 92.7 GPa vs. 93.2 GPa, obtained from the nano- and macro-IIT, respectively, which correspond to 3.78 vs. 3.8 GPa for H_R_. As the matching of the LSR and H_R_ at the nano- and macroscales was very good, we assigned both parameters as being ISE-free. Both the H_IT_ and H_R_ parameters were much larger in the FZ than in the BM. As the FZ and BM received the same initial heat treatment (before welding), the increased hardness of the FZ, compared to the BM, must be ascribed to the pulsed LBW of the joint (after its slicing). The nanoindents had been designed safely close to each other to permit the accurate tracing of the HAZ width on a mechanical basis. This is apparent in Figure 7a,b, where a drop can be observed in H_IT_ (from 6 to 5.5 GPa) and in H_R_ (from 4.25 to 4 GPa). The width of such a transition is uniform along the thickness of the weld seam and amounts to about 0.3 mm (for the selected plot mesh setting [43]). The width of the HAZ is more precisely detected at each relevant region of the weld seam, where all of the regions are in the 0.12–0.35 mm range (Figure 8). The green color in the color bar of Figure 7 corresponds to the hardness of the BM, which is 5.07 GPa in H_IT_ and 3.78 GPa in H_R_. Various coarse carbides can be observed at both of the lateral sides of the FZ near its interface. A few similar carbides, which had previously been identified as Ti-Ta carbides by means of electron diffraction spectroscopy [9], were also detected by means of SEM near the FZ (Figure 4b). The hardness of coarse carbides may reach quite high values [28,29], and it can markedly differ from those exhibited by fine carbides immersed in the refined γ-matrix of the FZ. We observed similar peak hardness values in the BM near the coarse carbides (Figure 7a,b).

### 3.3. Mechanical Performance of TR, NR, and BR

The FZ, HAZ, and BM were investigated in three distinct regions, that is, TR, NR, and BR, along the sample’s thickness and are indicated with the three green dashed rectangles and associated with indentation lines 1, 3 and 5, respectively, in Figure 3a. It should be recalled that each line has 21 groups of indentations, each of which contains five indentations. Each group is represented by its mean value of H_R_ and H_IT_. These two hardness parameters are shown as functions of the transversal (Y) direction in the three regions in Figure 8.

The ISE factor that affects H_IT_ can be estimated in more detail in the three relevant regions through the H_IT_-to-H_R_ ratio, denoted as C_ISE_. C_ISE_ was nearly constant (≈1.42) for all three regions and for the FZ, HAZ, and BM, despite the different penetration depth dependence of the two hardness parameters (i.e., h_m1_ versus h_m2_). Surprisingly, the two parameters scale one another, despite their different individual sensitivities to superficial and bulk anomalies upon indentation. Division of H_IT_ by the C_ISE_ factor gives H_IT,norm_, which adds further insights once such normalization is extended to the corresponding parameters in the TR, NR and BR. Figure 8 depicts the three parameter profiles at the TR, NR, and BR. The macrohardness, H_R_, in the BM (H_R,BM_ = 3.8 GPa) was taken as a reference and drawn as a violet dotted line. It is shown that the H_R_ and H_IT,norm_ profiles compare well with each other in all the regions, including in the HAZ. Hardness, H_R_, decreases from the FZ to the BM, with a steeper drop (~0.6 GPa) across the HAZ, thereby confirming the enhanced hardness attained after rapid solidification of the melt pool upon pulsed LBW of the IN792 DS alloy. Conversely, the hardness across the FZ declines slightly toward the weld centerline (Y = 0 mm). A similar sudden slope in the hardness profile can be observed in the three regions, which can be ascribed to changes in the microstructure (e.g., nanoscopic γ′ and carbide precipitates and crystal anisotropy) but only slightly to (compressive) long-range RS. Moreover, the H_R_ profiles at the TR, NR, and BR intersect the reference (horizontal line) H_R,BM_ at one common point, which corresponds to the outer boundary of the HAZ. This is useful for mechanically defining the HAZ width, and vice versa, the left boundary of the HAZ may be taken to be located where the hardness starts to drop outside the FZ. The HAZ widths are 0.15 mm at the TR, 0.35 mm at the NR, and 0.12 mm at the BR. These values are consistent with the results of a previous study [9] obtained using SEM images. The general tendencies of H_IT,norm_ ≥ H_R_ across the FZ and H_IT,norm_ ≤ H_R_ across the BM deserve attention. Moreover, the H_IT,norm_ line intersects the H_R_ line within the traced metallurgical HAZ. This is clearer in the TR and NR and less so in the BR, where more severe microstructure heterogeneities can be expected. Specifically, H_IT,norm_ and H_R_ tend to oscillate along the Y-directions in the BR, from the FZ to the BM, through the HAZ.

**Figure 9:**
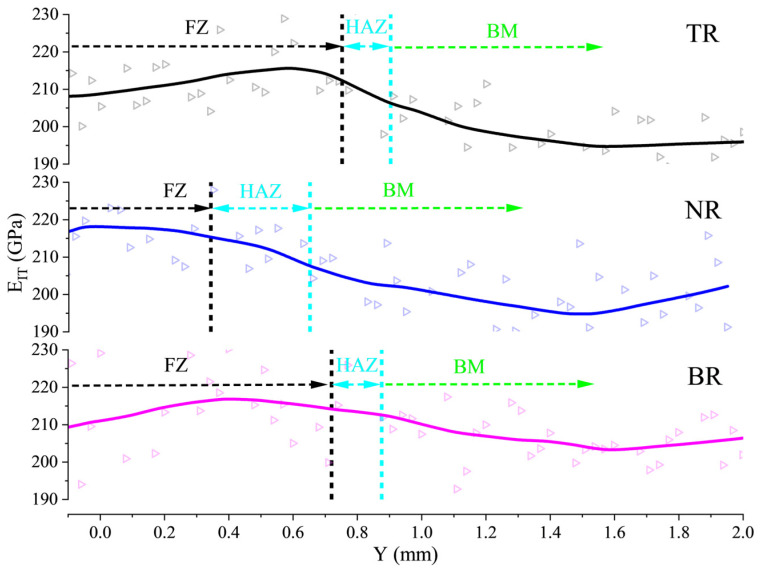
The measured E_IT_ profiles at the TR, NR, and BR. The black- and cyan-colored vertical dashed lines delimit the boundaries of the FZ (black dashed reference) and HAZ (cyan dashed reference) of the weld seam. The triangle symbols denote individual experimental points, and the thick lines are smoothed averages passing through them. The BM (green dashed reference) is also shown.

Figure 9 shows similar profiles for the E_IT_ vs. *Y*-axis displacement in the three relevant regions. The Δ symbols indicate the experimental values. The HAZ zones in the three regions replicate those of Figure 8. The thick lines represent the smoothed curves that pass through the data points, as computed by the *Lowess smoothing* function of the MATLAB 2024 software [42]. The indentation modulus profiles qualitatively mimic the hardness profiles shown in Figure 8, except for some small portions of the FZ, in which they either decrease or increase. The TR and BR in the FZ show a stiffness peak inside the FZ located near the FB, with a notable decrease in stiffness (toward the weld centerline) along the Y-direction, whereas it is located near the centerline at the NR, with a slight drop toward the FB. The indentation modulus progressively declines from the HAZ to the BM in a similar slope in each region until a common minimum (located between 1.4 and 1.6 mm) is reached. Away from this minimum (i.e., inside the BM), the E_IT_ again starts to increase slightly.

**Figure 10:**
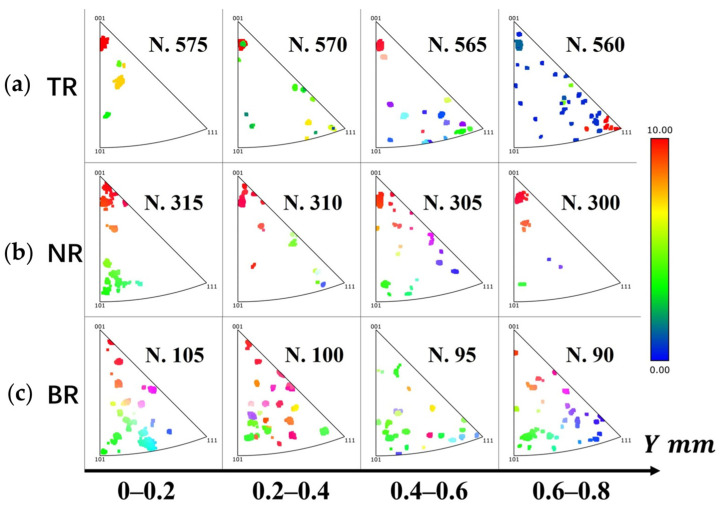
IPF//Z in the FZ at the (**a**) TR, (**b**) NR, and (**c**) BR.

Figure 10 compares the IPF along the positive *Y*-axis in the three regions. These EBSD results can help to shed light on the possible dependence of the crystal orientation on the E_IT_. Figure 10 should be analyzed together with Figure 5c–e. Figure 10a shows a strong <001> fiber at the TR, in group N.575, which is located near the weld centerline. This fiber effect tends to diminish with increasing distance from the weld centerline. Two orientations (<111> and <001>) are active in the last group, N. 560, which is in the FB, together with other minor orientations, and these reduce the texture effect. Moreover, the <001> and <101> orientations are both dominant in the NR, close to the weld centerline (N. 315), but their effect diminishes as the distance increases along the *Y*-axis. The original <001> orientation again becomes dominant in the last block (N. 300) in the BM. Conversely, several crystal orientations are involved at the BR, but none of them are dominant. In summary, Figure 10 confirms that the fiber effect partly explains the observed change in the E_IT_ but not that in H_IT_, shown in Figure 8 and Figure 9 respectively, and that both are dystonic parameters, meaning that more than one factor may explain their variation along the *Y*-axis direction. Hence, only two indentation parameters cannot explain the complexity of the performance change across the investigated weld seam.

### 3.4. Statistical Deconvolution Results

Efforts have been made to derive the phase composition of the FZ with alternative methods to microscopy, such as the statistical deconvolution method. This approach has been successfully proposed and applied by other authors [14,36]. In this work, we used minimal microstructure information from previous SEM inspections of the FZ [9], which revealed the presence of a γ′ (ordered intermetallic) phase, and Ti-Ta carbides embedded in the γ-matrix. Moreover, the authors of [27] found, in a different work, that the starting 70% of γ′ in the initial IN792 DS alloy reduced to ~24% of very fine spherical γ′ particles across the FZ versus ~26% after EBW of an identical IN792 DS alloy under the same prior heat treatment, preheating temperature, and heat input conditions. Conversely, Spiller et al. [41] did not find any γ′ in the FZ resulting from high rapid cooling. However, as those authors could not detect the eutectic phase, here we consider it to be irrelevant from the mechanical response point of view. Such prelaminar knowledge of the FZ microstructure helped us define our initial guess in the iteration process with reference to the nature and number of relevant phases contributing to the measured mechanical response in the FZ. These phases were porosity, the γ matrix, and both nanoscopic γ′ and carbide precipitates. For convenience, both γ′ and carbide phases were combined into one single phase, as both induce similar strengthening mechanisms in the FZ under the same thermal cycling conditions. However, as the amount of these carbides is expectably low compared to that of γ′, their individual mechanical effect on the γ′ phase may be irrelevant.

**Figure 11:**
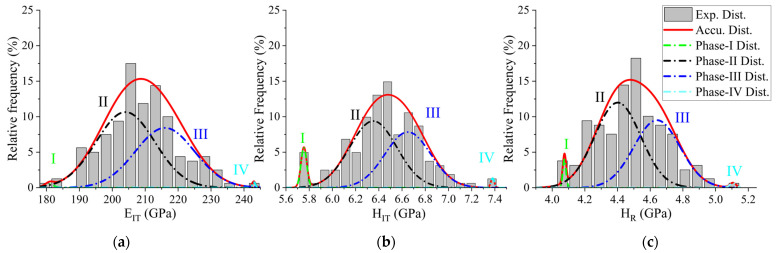
The Gaussian multimodal distribution (dash–dot lines) of the (**a**) E_IT_, (**b**) H_IT_, and (**c**) H_R_ resulting from the statistical deconvolution process applied to the entire set of experimental data inside the FZ of the three indentation parameters. The area subtended by each distribution provides an estimate of the phase fractions. The detected phases correspond to the pore phase (phase I), the γ matrix (phase II), the γ′/carbide precipitate phase (phase III), and the γ-matrix with a superimposed grain orientation effect and/or stronger local effect from the carbide phase (phase IV).

Statistical deconvolution was performed by trial and error on the four most likely representative peaks in the experimental spectra of each independent indentation parameter. As H_IT_ and H_R_ are dependent parameters, only one of them must be considered in the iteration process. The statistical model started with a *predictor* step, based on the selected peaks relative to one parameter, to build a theoretical density function that would best fit the experimental spectra. It then proceeded with a deconvolution step, which assigned the characteristic volume fraction, $f_{k}$; its associated mean value, $\mu_{HIT,k}$; and the standard deviation, $\sigma_{HIT,k}$, in a random statistics sense, to each selected k phase. This procedure was then repeated for the E_IT_ spectra as a *corrector* step. The iteration procedure converged when the deviation between the predicted phase fractions and the corrected ones was less than 5%. Otherwise, a new iteration restarted, with a different choice of selected peaks in the spectra, whenever 5% was not reached. The goodness of fit between the accumulative theoretical frequency density function (the red line in Figure 11) and the experimental spectra (bar plot) was expressed in terms of the R^2^ parameter (=1, very good). In this work, the multi-peak fitting function of the Origin 2022 software [44] was used for the statistical deconvolution of the experimental results (considering a constant bin width and subsequent normalization).

To validate the statistical deconvolution analysis, we decided to perform it in parallel over the entire FZ (*overall-FZ*) and separately over the individual regions (*TR-FZ*, *NR-FZ*, and *BR-FZ*) of the weld seam. In the former case, we used the entire parameter dataset, and in the latter we used the individual pertinent smaller subsets. Two major phases (phase II and phase III) and two minor phases (phase I and phase IV) were identified from both analyses. The main features of the two major phases are reported in Table 3. The minor phase characteristics are described separately later on in this section.

Figure 11 shows the goodness of the best fit between the *overall-FZ* empirical frequency density (histograms) and the modeled theoretical probability density function (in red) expressed by the R^2^ parameter, that is, 0.86, 0.89, and 0.85 for E_IT_, H_IT_, and H_R_, respectively. The phase fractions (obtained from E_IT_, H_IT_, and H_R_) were estimated to be within a tolerance of 1%, except for phase II at the *BR-FZ*, for which the tolerance was 4%. The two subtended normal distributions (blue dashed line, Figure 11) resulted from the *overall-FZ* statistical deconvolution. However, the smaller subset spectra may inevitably introduce larger errors and uncertainty into the estimation process, as shown by the presence of pairs of mean values and standard deviations in Table 3 (see ranges separated by – symbols). Pairs of this type exhibit two normal distributions [14]. We attempted to combine both distributions to finally match the fraction of either phase II or phase III that we found in the entire domain *overall-FZ*. From the *overall-FZ* analysis we estimated for phase II approximately 204 ± 9.0, 6.4 ± 0.2, and 4.4 ± 0.1 GPa for E_IT_, H_IT_, and H_R_, respectively. Identically, for phase III we estimated a volume fraction of 44% and 216 ± 9.0, 6.7 ± 0.2, and 4.6 ± 0.1 GPa for E_IT_, H_IT_, and H_R_, respectively.

Regarding the individual regions (*TR-FZ*, *NR-FZ*, and *BR-FZ*), we found the values listed in Table 3. It is worth noticing that upon merging of the two distinct normal distributions the estimated phase fractions in the three regions compared well with those estimated for the *overall-FZ*, since any deviation was always lower than or equal to 5%. This supports the self-validation of the statistical model, even for smaller data subsets. Nevertheless, greater deviation of the phase fraction was found at the *BR-FZ* for phase II. In this region, it is not clear whether the deviation was attributed to true and significant microstructure heterogeneity/anisotropy or to poor statistics. The double-value distribution of E_IT_ for phase II in region *BR-FZ* underlines that this phase might include two similar phases with close values for E_IT_ (201 ± 2.0 GPa and 215 ± 1.2 GPa), which are consistent with the single mean value of E_IT_ (207 ± 3.2 GPa) for region *TR-FZ* and (211 ± 3.7 GPa) for region *NR-FZ*, thereby confirming that the stiffness of the matrix for the *overall-FZ* was 206 ± 9.0 GPa. Thus, due to the consistency between stiffness and hardness, we can finally determine that *BR-FZ* was softer than *TR-FZ* and *NR-FZ*, whereas *NR-FZ* was harder than *TR-FZ* (with respect to both H_IT_ and H_R_).

The remaining phases, I and IV, physically represent the soft/compliant and hard/stiff phases. These are located at the two ends of the spectra (Figure 11). The indentation hardness of phase I is ~5.7 GPa, and its indentation modulus falls within the 180–190 GPa range. Conversely, phase IV presents ~7.4 and ~241 GPa for H_IT_ and E_IT_, respectively. The estimated phase fractions were ~2% and 3% at *TR-FZ* and *BR-FZ*, respectively, for phase I, but the fraction was not clearly discerned at *NR-FZ*. This means that accidental porosity was favored at *TR-FZ* and *BR-FZ* but not at *NR-FZ*. The estimated phase IV fractions were ~2% at *TR-FZ*, ~6% at *NR-FZ*, and varied over the 2–3% range at *BR-FZ*.

## 4. Discussion

Reliable measurements of the mechanical performance of the pulsed laser welded IN792 DS alloy are paramount in assessing their integrity and their safe function in service. However, the current standard guidelines [35] recommended to extract the indentation parameters cannot be applied to materials that initially contain RS. Moreover, the indentation hardness suffers from an ISE effect which limits quantitative comparisons between indentation hardness values at different penetration depths in both the micro- and nanoscale range. In this work, a new IIT post-processing methodology has been proposed for the extraction of a new ISE-free hardness parameter (H_R_). It has been derived from the loading secant stiffness rate (LSR) parameter via the more insightful secant stiffness versus h plot. The H_R_ parameter complements the two well-known indentation parameters, i.e., H_IT_ and E_IT_, to determine the local bulk mechanical response of arbitrary RS-affected components while allowing accurate benchmarks (material, process, and part geometry and testing conditions) to be determined. Furthermore, as the microstructure composition of welded nickel-based superalloys is generally difficult to detect with microscopic methods, a statistical deconvolution method has been developed. In this study, such a computational approach enabled the estimation of the most relevant phases composing the investigated IN792 DS alloy welded by pulsed LBW. In this study, as the (i) mechanical performance based on the new indentation parameters could not directly be compared with counterparts in the literature, (ii) the standard H_IT_ parameter was affected by the ISE problem and the (iii) E_IT_ parameter was strongly influenced by RS, being in turn depending on joint geometry, materials, welding conditions, etc., such that precise and quantitative comparisons were not possible. To the authors’ knowledge, no previous studies have been reported on the welding of the IN792 superalloy by pulsed LBW with special reference to nano-IIT measurements of indentation hardness or stiffness of nanocrystalline precipitates (in the FZ).

Thus, analyses based on consideration of the λ factors of alloys analogous to the one investigated here, such as CMSX-4 and CM 247A, could be attempted. This factor considers the ratio of each indentation property (H_IT_ and E_IT_) of the precipitate to that of the matrix. According to the literature, λ_E_ must lie in the range of 1.05–1.09, and its variation typically originates from a crystal orientation [20].

### 4.1. Benchmark Based on Indentation Modulus and Hardness from the Literature

In this study, the integral combination of the nano-IIT, carried out on the pulsed IN792 DS alloy welded by pulsed LBW, and the statistical deconvolution method applied to the *overall-FZ* returned E_IT_ and H_IT_ values equal to 204 ± 9.0 and 6.4 ± 0.2 GPa for the γ matrix (phase II) and to 216 ± 9.0 and 6.7 ± 0.2 GPa for the γ′/carbide precipitates (phase III). The ratios of λ_E_ and λ_H_ measured here were 1.06 and 1.05 respectively.

Durst and Göken [45] reported variable hardness ratio (λ_H_) and indentation modulus (λ_E_) values in the range of 1.20–1.70 and 1.10–1.20 due to different concentrations of refractory elements in CMSX-4, CMSX-6 and CMSX-10 alloys. Gan et al. [46] reported slightly different ranges for these two parameters, namely, 1.01–1.05 and 1.05–1.10, for CMSX-4. Similarly, M. Kalyan et al. [47] provided an λ_E_ value of ~1.04 for CM 247A. The ratios computed here were smaller than those reported in the literature [45], but the consistency found with [46] and [47] was probably due to the size of the precipitates. However, λ_H_ can vary significantly from 1.9 to 1.05 due to different penetration depths (25 nm to 175 nm) [48].

More reliable and pertinent experimental validations require well-designed self-contained IIT experiments that systematically vary the welding alloys and parameters and testing conditions. It is expected that the use of the new ISE-free hardness parameter, H_R_, will accelerate the validation process, as it will in general enhance comparability even in the presence of RS.

### 4.2. Effect of Phase Fraction on Indentation Parameters

As can be seen in Table 3**,** the *BR-FZ* shows the lowest level of hardness and, in general, quite large standard deviations out of all the mechanical parameters. Pan et al. [11] reported that the melt pool in the lower section of the weld seam experienced the highest cooling rate and severe fluctuations during fully penetrated LBW (keyhole mode), resulting in lower volume fractions of precipitates. The cooling rate profiles computed with our FE-based LBW model for identical alloys, geometries and welding conditions also confirm this thermal circumstance at the *BR-FZ*. Conversely, the *TR-FZ* exhibited a comparatively lower cooling rate, which promoted interdendritic microsegregation (Mo, Si, and Mn), a longer diffusion time, and, consequently, a larger fraction of carbides supported by a relatively large hardness index. The drop in stiffness in the *TR-FZ* of phase III may have resulted from the competition between the decreasing cooling rate effect (which enhanced the local stiffness via the formation of nanoscopic γ′ and carbide precipitates) and the loss in stiffness of columnar grains. The latter tended to equalize the <001> orientation while approaching the weld centerline, as shown in Figure 5a. The high level of hardness of the nanoscopic (Ti and Ta) carbides and the γ′ round particles partially explains the larger indentation parameters in the FZ [49] in comparison to the HAZ and BM. Gruber et al. [28] reported the presence of NbC and TiC carbides increased the hardness of nickel-based superalloys by as much as 50 HV (or 0.53 GPa). These microstructure effects were driven by overlaying nearly uniform compressive long-range RS (Y-direction) components which emerged after sample slicing, hence ensuring a generalized stiffer welded seam than the BM and HAZ.

**Figure 12:**
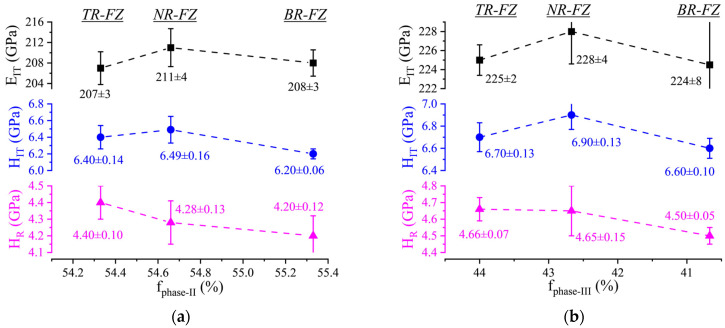
Relationship between indentation parameters, H_R_, (pink triangle), H_IT_ (blue circle), E_IT_ (black square) at the three relevant regions of the FZ (TR, NR, BR) and phase fraction present for (**a**) phase II and (**b**) phase III after statistical deconvolution analysis. For simplicity, double-value distributions have been converted into one single-value distribution [14].

Figure 12 summarizes in a pictorial form the trend of the mechanical performance in terms of the three measured (mean value) indentation parameters versus the estimated phase fraction (phase II) of the matrix and that of the second phase (phase III) at the three regions within the FZ (TR-FZ, NR-FZ and BR-FZ). The profiles of E_IT_ and H_IT_ versus both phase fractions (phase II in Figure 12a and phase III in Figure 12b) show a maximum peak at *NR-FZ*. This is opposed to the H_R_ profiles, which underlines a strong monotonic trend against the phase fractions of phases II and III. The H_R_ index shows a more intimate link with microstructure than both E_IT_ and H_IT_ do, which in turn highlights their overlaying dependence from other factors than microstructure, such as a long-range RS state.

### 4.3. Effect of Residual Stress

Recent studies on continuous milling and cutting operations on RS-affected components have shown that internal RS may even revert from a tensile to a compressive state [50]. Moreover, numerical predictions from a domestic finite element-based LBW model which mimicked the thermo-mechanical behavior of the investigated LBW IN792 DS joint (to be published), supported by Neutron diffraction measurements [51], have confirmed similar RS relaxation upon progressive slicing of the initial welded joint until the investigated sample was achieved. As the prominent tensile long-range RS originally developed along the *X*-axis tends to progressively relax upon slicing, the transversal (Y-direction) component of the compressive long-range RS remains locked over the Y-Z surface of the indentation sample. On the other hand, the short-range RS, because of the intimate bonds in the microstructure, is likely to remain unaltered after slicing, though slightly altered during the mechanical preparation of the sample. However, the latter stress contribution may result in a negligible effect on the indentation parameters when a relatively high indentation load (in the nanoscale range) is selected, as in this study. Additionally, the direct measurement of long-range RS is beyond the primary goal of this study; it is because of the susceptibility to relaxation during indentation that a valuable mechanical performance index is to be considered in the engineering design of a welded joint. All the measured E_IT_ values at the BM were always above the expected Young’s modulus of the IN792 DS alloy, that is, 170 GPa [52]~180 GPa [53]. It is well known that E_IT_ is particularly sensitive to RS, and it becomes larger than the Young’s modulus for compressive RS and smaller for tensile RS [54]. As the compressive transversal RS state is almost uniform across the FZ, it should not affect the spatial distribution of the measured indentation parameters, which, instead, are influenced by changes in grain orientation.

The results collected so far suggest that when all three of the indentation parameters are syntonic, i.e., all three are large or all three are small, the local mechanical performance can be uniquely determined. However, if one of the three parameters is dystonic, the local mechanical response is uncertain and a more accurate three-dimensional evaluation of the RS distribution is required. However, as slicing may dramatically alter the original self-balanced 3D RS state introduced by the welding process, from sample to sample [51], judicious choice of the cutting procedure is recommended, bearing in mind that this may reduce comparability among different studies. More importantly, in the case of macro-IIT, such remnant RS may accidentally relax during testing, resulting in severe compliance problems and consequently unreliable indentation parameters [55].

## 5. Conclusions

The mechanical performance and the phase composition of the FZ of an (EDM) sliced butt-welded joint upon pulsed LBW of an IN792 DS alloy has been determined at three relevant regions of the hourglass-shaped weld seam by the combination of a nanoindentation mapping strategy and a statistical deconvolution analysis. A final correlation between the estimated phase proportions and the mechanical performance has also been attempted. Upon sample slicing, the indented (Y-Z) surface, especially at the FZ and HAZ, inherited an overlaying uniform compressive RS state from the Y-direction component of the original 3D stress state imparted by the LBW process. This resulted in a higher indentation modulus than the expected Young’s modulus at those zones. The new rate-based hardness (H_R_) exhibits a unique ISE-free feature that enables quantitative comparisons of the measured mechanical performance to virtually be made for any selected peak load for different microstructures, RS or texture (if any), and thus among different nickel superalloys, LBW parameters, welding processes, initial heat treatments, etc. Both the H_IT_ and H_R_ parameters were much larger in the FZ than in the HAZ and BM. The hardness parameters of the BM were 5.07 ± 0.16 GPa for H_IT_ and 3.78 ± 0.10 GPa for H_R_, whereas the indentation modulus was 203 ± 11 GPa. The syntonic behavior of the three indentation parameters permitted a univocal discrimination to be made of the mechanical performance in the presence of RS and texture effects.

A constant ISE ratio parameter (C_ISE_ = 1.42) was found to be characteristic of the entire weld seam. This enabled H_IT_ to be used, similarly to H_R_, for benchmarking the mechanical performance with other studies. The high resolution of nano-IIT provided a mechanically based estimation of the HAZ width, a result that can be useful when metallographic means are prone to fail. The HAZ experienced a dramatic drop in hardness (~0.6 GPa for H_R_ and ~0.9 GPa for H_IT_) and stiffness (~6 GPa). The EBSD analysis revealed quite different crystal orientations of the microstructure in the upper and lower sections of the weld seam, resulting from unique thermal conditions that accompanied the fully penetrated LBW. A <001> crystal orientation dominated the upper section, whereas a <101> orientation grain structure prevailed in the lower section. In the FZ, the former was characterized by a low cooling rate during solidification, which led to a large amount of much harder precipitates (round γ′ particles and carbide precipitates), whereas the latter was affected by a much higher cooling rate, a reduced amount of nanoscopic precipitates, and greater heterogeneity in the microstructure and crystal orientation.

The absolute values of the indentation parameters outside the FZ were primarily ascribed to the change in the transversal (Y-direction) of the long-range RS, whereas the relative change reflected the grain orientation (<001>) effect.

**Figure 13:**
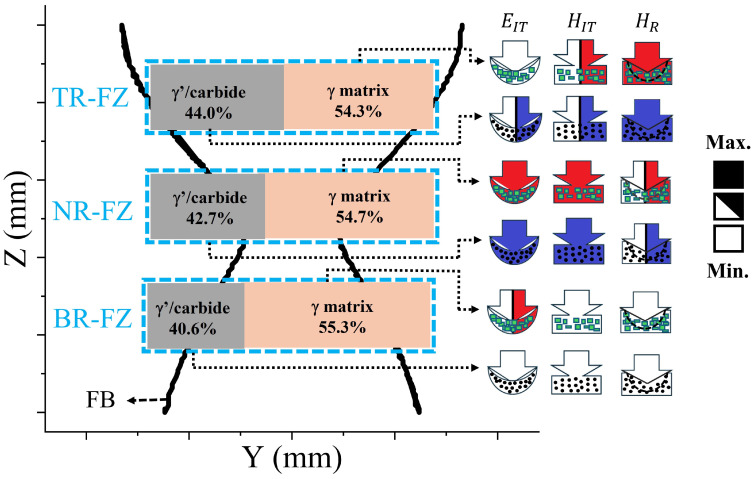
Schematic summary of the phase proportions in three investigated regions (*TR-FZ*, *NR-FZ* and *BR-FZ*) of the FZ with the associated mechanical performance expressed in terms of the three indentation parameters (E_IT_, H_IT_ and H_R_). The fully colored symbols represent the highest values, and vice versa; for each region, the matrix, γ, has been filled with (green) rectangular blocks and the second phase (a mixture of nanoscopic γ′ and carbide precipitates) with (black) dots. The more penetrating arrow in the HR symbol denotes the bulk ISE-free parameter, and vice versa for the ISE-affected H_IT_ parameter, while the E_IT_ parameter is sketched with a complying sample under the indentation. The degree of filling of the arrows indicates the largest (fully filled), mid (semi-filled), and lowest (unfilled) values.

A statistical deconvolution analysis was conducted to discern the mechanical performance ranges associated with the four phases present in the FZ of the pulsed LBW IN792 DS alloy, and the individual phase fractions were estimated by an iteration procedure based on the two independent indentation parameter spectra. The convergence of such iterations led to four relevant phases with the associated characteristic indentation properties which have pictorially been summarized in Figure 13 and quantitatively as follows: phase I (soft/compliant), phase IV (hard/stiff), phase II (γ-matrix), and phase III (nanoscopic γ′ round particles and carbides); the coarse matrix in the FZ amounted to 54% and was associated with approximately 209 ± 9.0, 6.4 ± 0.2, and 4.4 ± 0.1 GPa, whereas the second phase amounted to 44% and was associated with 216 ± 9.0, 6.7 ± 0.2, and 4.6 ± 0.1 GPa for E_IT_, H_IT_, and H_R_, respectively.

Further studies which will take into account different sample geometries, alloys, joint configurations and residual stress distribution are needed to confirm the validity of the proposed IIT methodology based on ISE-dependent H_R_ and LSR parameters. A follow up of this work, will focus on the thermomechanical behaviour of the investigated DS IN792 butt-welded joint, using a domestic finite element pulsed LBW model, to confirm the validity of the phases and associated indentation property ranges estimated via deconvolution analysis at the three relevant regions of the FZ.

## Figures and Tables

**Table 1:** Nominal chemical composition of IN792 DS [9].

Element	Ni	C	Al	Ti	Cr	Co	Mo	W	Ta
at.%	Bal.	0.39	8.88	4.46	14.07	9.13	1.16	1.56	1.46

**Table 2:** Process parameters of the pulsed LBW butt joint in IN792 DS [9].

*T_pre-heating_* [°C]	*P* [W]	*v_scan_* [mm/s]	Welding Mode
200	$P_{pk}=2080$ $P_{ave}=1250$ f=60 Hz	25	Pulsed line

**Table 3:** Summary of the indentation parameters for each detected phase (phase II and phase III) resulting from the statistical deconvolution process across the entire (overall) FZ and in the three individual regions (top, neck, and bottom). Phase II and phase III correspond to the main phases detected in the FZ, namely, the γ-matrix and the γ′/carbide precipitate phase, respectively.

Location	Statistical Data	Phase II			Phase III		
		E_IT_	H_IT_	H_R_	E_IT_	H_IT_	H_R_
Overall-FZ	μ (GPa)	204	6.4	4.4	216	6.7	4.6
	σ (GPa)	9.0	0.2	0.1	9.0	0.2	0.1
	f (%)	55	53	55	44	43	44
TR-FZ	μ (GPa)	207	6.3–6.5	4.4	221–229	6.6–6.8	4.6–4.7
	σ (GPa)	3.2	0.04–0.1	0.1	1.2–0.4	0.03–0.1	0.03–0.04
	f (%)	54	55	54	44	43	45
NR-FZ	μ (GPa)	211	6.4–6.5	4.1–4.4	222–234	6.7–7.1	4.5–4.8
	σ (GPa)	3.7	0.06–0.1	0.03–0.1	2.4–1.0	0.1–0.03	0.1–0.05
	f (%)	56	53	55	44	41	43
BR-FZ	μ (GPa)	201–215	6.2	4.1–4.3	221–228	6.4–6.8	4.5
	σ (GPa)	2.0–1.2	0.06	0.04–0.08	6.0–2.0	0.05–0.04	0.05
	f (%)	53	54	59	40	42	40

μ: mean value of the distribution. σ: standard deviation. f: fraction of each integral area of the distribution to the total area.

## Data Availability

The original contributions presented in this study are included in the article. Further inquiries can be directed to the corresponding author.

## Abbreviations

The following abbreviations are used in this manuscript:

BM	Base material
BR	Bottom region
BR-FZ	Fused zone in the bottom region
BSE	Backscattered electrons
DS	Directionally solidified
EBSD	Electron backscatter diffraction
E_IT_	Indentation modulus
FB	Fused boundary
FZ	Fused zone
HAZ	Heat-affected zone
h_c_	Contact depth
hm1	Maximum penetration depth on loading
hm2	Maximum penetration depth at the holding stage
H_IT_	Indentation hardness
H_IT,norm_	Normalization of indentation hardness
H_R_	Rate hardness, nearly-ISE-free hardness based on LSR parameter
IC	Indentation curve
IIT	Instrumented indentation test
ISE	Indentation size effect
LBW	Laser beam welding
LSR	Loading secant stiffness rate
NR	Neck region
NR-FZ	Fused zone in the neck region
RS	Residual stress
SEM	Scanning electron microscopy
S_u_	Unloading stiffness
TR	Top region
TR-FZ	Fused zone of top region
